# CD14-positive extracellular vesicles in bronchoalveolar lavage fluid as a new biomarker of acute respiratory distress syndrome

**DOI:** 10.1152/ajplung.00052.2022

**Published:** 2022-03-02

**Authors:** Rahul Y. Mahida, Joshua Price, Sebastian T. Lugg, Hui Li, Dhruv Parekh, Aaron Scott, Paul Harrison, Michael A. Matthay, David R. Thickett

**Affiliations:** ^1^Birmingham Acute Care Research Group, Institute of Inflammation and Ageing, University of Birmingham, Birmingham, United Kingdom; ^2^Institute of Inflammation and Ageing, University of Birmingham, Birmingham, United Kingdom; ^3^Department of Anesthesia and Critical Care, The Second Affiliated Hospital and Yuying Children’s Hospital of Wenzhou Medical University, Wenzhou, China; ^4^Cardiovascular Research Institute, Departments of Medicine and Anesthesia, University of California, San Francisco, California

**Keywords:** acute respiratory distress syndrome, extracellular vesicles, monocyte, sepsis

## Abstract

Recent studies have indicated that extracellular vesicles (EVs) may play a role in the pathogenesis of acute respiratory distress syndrome (ARDS). EVs have been identified as potential biomarkers of disease severity and prognosis in other pulmonary diseases. We sought to characterize the EV phenotype within bronchoalveolar lavage (BAL) fluid of patients with ARDS, and to determine whether BAL EV could be used as a potential biomarker in ARDS. BAL was collected from patients with sepsis with and without ARDS, and from esophagectomy patients postoperatively (of whom a subset later developed ARDS during hospital admission). BAL EVs were characterized with regard to size, number, and cell of origin. Patients with sepsis-related ARDS had significantly higher numbers of CD14^+^/CD81^+^ monocyte-derived BAL EV than patients with sepsis without ARDS (*P* = 0.015). However, the converse was observed in esophagectomy patients who later developed ARDS (*P* = 0.003). Esophagectomy patients who developed ARDS also had elevated CD31^+^/CD63^+^ and CD31^+^/CD81^+^ endothelial-derived BAL EV (*P* ≤ 0.02) compared with esophagectomy patients who did not develop ARDS. Further studies are required to determine whether CD31^+^ BAL EV may be a predictive biomarker for ARDS in esophagectomy patients. CD14^+^/CD81^+^ BAL EV numbers were significantly higher in those patients with sepsis-related ARDS who died during the 30 days following intensive care unit admission (*P* = 0.027). Thus, CD14^+^/CD81^+^ BAL EVs are a potential biomarker for disease severity and mortality in sepsis-related ARDS. These findings provide the impetus to further elucidate the contribution of these EVs to ARDS pathogenesis.

## INTRODUCTION

Acute respiratory distress syndrome (ARDS) is a hyperinflammatory pulmonary disorder, which most commonly develops following sepsis. Neutrophilic inflammation and alveolar-capillary barrier damage lead to alveolar edema and refractory hypoxia, requiring prolonged mechanical ventilation (1). Mortality remains high at 35%–45% (2). Survivors have long-term morbidity and increased risk of developing lung fibrosis. There is an urgent need to identify novel therapeutic targets for ARDS, especially due to the evolving SARS-CoV-2 pandemic in which ARDS is the main cause of mortality (3).

Extracellular vesicles (EVs) are membrane-bound anuclear structures that constitute an intercellular communication mechanism (4). EVs allow targeted transfer of diverse biological cargo (including mitochondria, RNA, and cytokines) between different cell types (5). Uptake of EV can alter gene expression within host cells (6). The tetraspanins CD9, CD63, and CD81 are transmembrane proteins commonly expressed on EV, and can be used to isolate EV in acellular biofluids (7). There are two main subtypes of EV: exosomes and microvesicles. Exosomes are 30–150 nm in diameter and form by fusion of multivesicular bodies with the plasma membrane. Microvesicles are 100–1,000 nm in diameter and form by outward blebbing of the plasma membrane (4). With overlap in size and common markers (e.g., tetraspanins), differentiating exosomes and microvesicles is challenging, however endosomal sorting complex required for transport (ESCRT) proteins such as syntenin are candidate internal cargo markers specific to exosomes (8).

Recent evidence indicates that EV play an important role in ARDS pathogenesis by promoting persistent inflammation. Pathogenic EV are released when human ex vivo perfused lungs are injured with *Escherichia coli*; isolation of these EV and administration to uninjured human lungs can also induce inflammatory lung injury (9). Murine models of lipopolysaccharide lung injury have shown that EV transfer of microRNA cargo (e.g., miR-466) to alveolar macrophages can increase inflammatory cytokine release (10). Recent studies have indicated that CD14^+^ BAL EV may be a potential biomarker for disease activity in chronic obstructive pulmonary disease (COPD) (11).

We previously showed that alveolar macrophages isolated from patients with sepsis-related ARDS have impaired efferocytosis (ability to clear apoptotic cells) compared with control-ventilated patients with sepsis without ARDS (12). Alveolar macrophage efferocytosis negatively correlated with alveolar neutrophil apoptosis, and bronchoalveolar lavage (BAL) cytokines IL-8 and IL-1α. Impaired efferocytosis was associated with increased 30-day mortality and duration of mechanical ventilation, indicating that this alveolar macrophage functional defect contributes to ARDS pathogenesis (12). Treatment of healthy alveolar macrophages with BAL from patients with ARDS also impairs efferocytosis and downregulates Rac1 expression (which causes cytoskeletal rearrangement allowing apoptotic cell engulfment), reproducing the same functional defect observed in ARDS (13). However, the causative immunomodulatory factors within BAL of patient with ARDS remain to be identified. Identification of differentially expressed EV populations within ARDS BAL may guide further investigation into the mechanism of this alveolar macrophage functional defect

In this study, the objective was to characterize the EV phenotype in the BAL of patients with sepsis-related ARDS and to compare this EV population to that in control patients with sepsis and in postesophagectomy patients (a subset of whom later developed ARDS). We also aimed to determine whether BAL EV could be utilized as a potential biomarker in sepsis-related ARDS.

## MATERIALS AND METHODS

### Ethical Approval

As previously described (12), ethical approval was obtained to recruit ventilated patients with sepsis, with and without ARDS (REC 16/WA/0169). Ethical approval was also obtained to recruit patients undergoing transthoracic esophagectomy for esophageal carcinoma (REC 12/WM/0092) in the VINDALOO trial (14, 15). For patients who lacked capacity, permission to enroll was sought from a personal legal representative in accordance with the UK Mental Capacity Act (2005). For patients with capacity, written informed consent was obtained from the patient.

### Patient Recruitment and Bronchoalveolar Lavage

Invasively ventilated adult patients with sepsis, with and without ARDS, were recruited from the intensive care unit of the Queen Elizabeth Hospital Birmingham, UK from December 2016 to February 2019 and BAL was collected as previously described, within 48 h of initiation of mechanical ventilation (12). Patient demographic and physiological details are in this prior publication. In the VINDALOO trial (15), patients underwent postoperative bronchoscopy and BAL following esophagectomy. Following unblinding of the study, those patients who received placebo were identified; BAL samples from the four patients who developed ARDS during their postoperative course and eight patients who did not develop ARDS were analyzed in this study. For all patients, sterile, isotonic saline (2 × 50 mL aliquots) was instilled into a subsegmental bronchus of the lingula or right middle lobe. Recovery ranged between 20 and 60 mL and did not differ between patient groups. BAL fluid collection was standardized across all patients in keeping with European guidelines (16). BAL fluid was rendered acellular by centrifuging at 500 *g* for 5 min, then stored at −80°C in aliquots before use in this study. Differential cell count was performed on cells isolated from BAL.

### BAL Extracellular Vesicle Characterization

A single-particle interferometric reflectance image sensing platform was used (Exoview R100 reader, NanoView Biosciences) to detect and characterize EV size, number, and surface marker expression within BAL samples (17). The Exoview platform allows quantification and phenotyping of EV ≥50 nm in diameter (7). EV numbers in BAL were measured using ExoView Tetraspanin kits (NanoView Biosciences) according to manufacturer’s instructions. Each spot on an Exoview Tetraspanin chip is an independent test, interacting with only a small volume surrounding itself. Accounting for volume and spot size, a normalized particle number can be generated and when multiplied by the dilution factor and a correction factor of 10^4^, data can be expressed as EV/mL. Each spot on an Exoview Tetraspanin chip has the capacity to bind up to 5,000 EVs before becoming saturated. According to the manufacturer’s instructions, at least a 1:1 dilution of any biofluid with incubation solution is required before incubation on Exoview Tetraspanin chips. Preliminary studies had found that use of dilutions lower than 1:20 resulted in chip saturation, with the number of EVs exceeding the chip binding capacity. BAL samples were therefore diluted 1:20 with incubation solution to prevent chip saturation. Diluted BAL (35 µL) was loaded on the ExoView Tetraspanin chips and incubated for 16 h in a sealed, humidified 24-well plate at room temperature to allow EV binding. Each chip contained spots printed with mouse anti-human antibodies against the tetraspanins CD63 (clone H5C6), CD81 (clone JS81), and CD9 (clone H19a) that enabled EV capture. Chips also contained spots with mouse IgG1κ matching isotype antibodies, used as a control for nonspecific EV binding. Chips were washed thrice with incubation solution; after each wash plates were shaken at 500 rpm (LSE digital microplate shaker, Corning). Chips were then stained with a fluorescent antibody cocktail for 1 h at room temperature in the dark to characterize EV surface markers. Fluorophore-conjugated mouse anti-human antibodies included: CD14-Allophycocyanin (1:100 dilution, clone 61D3, Invitrogen), CD206-Phycoerythrin (1:100 dilution, clone 19.2, Invitrogen), EpCam-AlexaFluor488 (1:400 dilution, clone 94 C, BioLegend), CD66b-AlexaFluor647 (1:200 dilution, clone G10F5, BD Biosciences), CD31-Phycoerythrin (1:100 dilution, clone WM59, Invitrogen), and CD41-AlexaFluor488 (1:400 dilution, clone HIP8, BioLegend). Chips were washed thrice again, with a final wash in distilled water, before being imaged using the ExoView R100 reader and nScan2 v2.76 software. Data were analyzed using NanoViewer v2.82, utilizing mouse IgG capture spots as negative control (indicative of nonspecific binding) for fluorescent gating, and EV sizing thresholds set at 50–200 nm diameter. EV cell origin data were correlated with previously recorded clinical outcomes and immune cell functional parameters (12). Data from each tetraspanin capture spot (CD63, CD81, and CD9) were calculated by subtracting negative control values from IgG spots.

### Statistical Analysis

Data were analyzed using Prism 8 software (GraphPad). Parametric data are shown as mean and standard deviation. Nonparametric data are shown as median and interquartile range. Differences between continuously distributed nonparametric data were assessed using Mann–Whitney tests. Differences between three or more unpaired nonparametric data sets were assessed using Kruskal–Wallis test by ranks. Two-tailed *P* values of ≤0.05 were considered significant. For all figures and tables, *n* represents the number of subjects in each group.

## RESULTS

The demographic and clinical cha racteristics of ARDS and control patients from the two cohorts analyzed are shown in Table 1. BAL samples were analyzed from 17 patients with sepsis-related ARDS, 14 patients with sepsis without ARDS, 4 esophagectomy patients who later developed ARDS, and 8 esophagectomy patients who did not develop ARDS.

**Table 1.** Patient demographic and clinical characteristics

	Patients with Sepsis		Esophagectomy Patients	
	With ARDS (*n* = 17)	Without ARDS (*n* = 14)	Developed ARDS (*n* = 4)	Did not Develop ARDS (*n* = 8)
Demographics	
Age in years	59.2 (13.9)	55.1 (16.3)	63.3 (6.9)	65.8 (7.9)
Male sex, *n* (%)	15 (71%)	11 (65%)	4 (100%)	4 (50%)
Ethnicity: Caucasian, *n* (%)	21 (100%)	17 (100%)	4 (100%)	7 (88%)
Smoking status, *n* (%)				
Current	7 (33%)	5 (29%)	1 (25%)	0 (0%)
Ex-smoker	9 (43%)	6 (35%)	1 (25%)	6 (75%)
Never smoker	2 (10%)	1 (6%)	2 (50%)	2 (25%)
Unknown	3 (14%)	5 (29%)	0 (0%)	0 (0%)
ICU LoS in days: median (IQR)	23.0 (12.8–33.8)	12.0 (7.5–19.0)	9.5 (5–22.3)	4.5 (2.5–7.8)
Hospital LoS in days: median (IQR)	33.0 (15.3–52.5)	24.0 (13.0–39.5)	22.0 (17.3–53.8)	12 (8.3–15.8)
Inpatient mortality, *n* (%)	7 (33.3%)	3 (17.6%)	1 (25%)	0 (0%)
BAL leukocyte count × 10^6^: median (IQR)	15.8 (7.4–31.3)	6.4 (3.8–27.0)	11.0 (5.9–18.6)	7.8 (2.1–10.5)
% Neutrophils in BAL	69.9 (21.5)	49.0 (30.6)	20 (20)	9.5 (10.1)

Values are means (SD) unless otherwise indicated. ARDS, acute respiratory distress syndrome; BAL, bronchoalveolar lavage; ICU, intensive care unit; IQR, interquartile range; LoS, length of stay.

**Figure 1.**
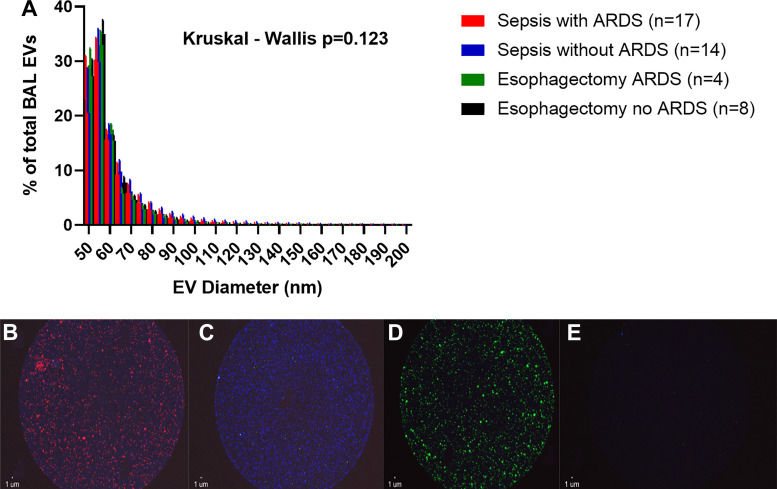
Bronchoalveolar lavage (BAL) extracellular vesicles (EV) size distribution and representative Exoview fluorescence images. *A*: BAL EV size distribution measured using single-particle interferometric reflectance image sensing (Exoview R100 platform). Statistical analysis by Kruskal–Wallis test, *n* ≥ 4 in each group. There was no significant difference in EV size distribution between patients with sepsis-related acute respiratory distress syndrome (ARDS), patients with sepsis without (ARDS), esophagectomy patients who developed ARDS, and esophagectomy patients who did not develop ARDS (*P* = 0.123). EV measured between 150 and 200 nm are not shown in this graph as these accounted for <1% of total EV population in all three groups. *B*–*D*: representative Exoview fluorescence images demonstrating EV surface marker expression. *B*: detection of CD66b^+^ EV (AlexaFluor647) on CD63 tetraspanin spot. *C*: detection of EpCam^+^ EV (AlexaFluor488) on CD9 tetraspanin spot. *D*: detection of CD31^+^ EV (Phycoerythrin) on CD9 tetraspanin spot. *E*: representative Mouse IgG spot negative control showing low nonspecific EV binding in BAL samples.

BAL EVs were characterized with regards to size, number, and surface marker expression to indicate cellular origin. The size distribution of BAL EVs did not differ across patient groups (Fig. 1*A*). Representative fluorescence images from the Exoview platform demonstrating EV surface marker expression and lack of nonspecific binding are shown in Fig. 1, *B*–*E*.

**Figure 2.**
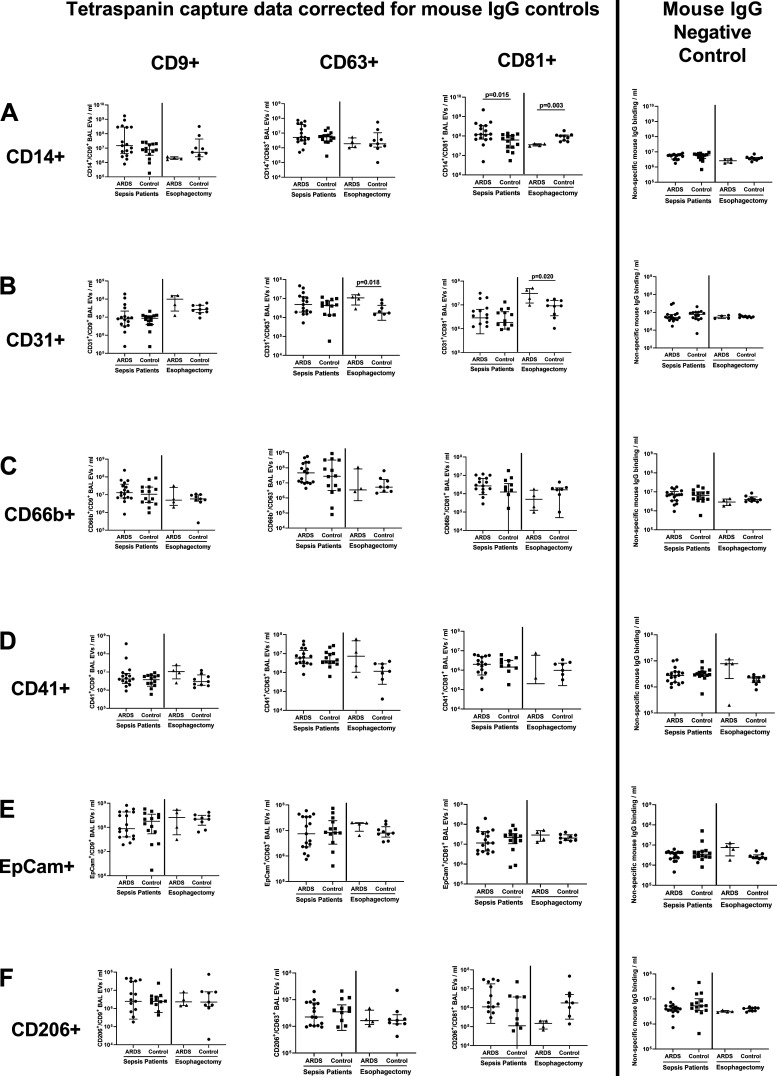
Bronchoalveolar lavage (BAL) extracellular vesicles (EV) surface marker expression in acute respiratory distress syndrome (ARDS) and control groups across sepsis and esophagectomy patient cohorts. BAL EV surface marker expression measured by tetraspanin antibody capture (CD9/63/81) and fluorescent antibody labeling via the Exoview R100 platform (EpCam for epithelial, CD66b for neutrophil, CD206 for alveolar macrophage, CD31 for endothelial, CD41 for platelet, and CD14 for monocyte origin). All data expressed as median and inter-quartile range, *n* ≥ 8 in each group. Statistical analyses by Mann–Whitney *U* tests. Fluorescence data are presented by individual tetraspanin capture spots. Data from each tetraspanin capture spot (CD9, CD63, and CD81—first three columns from the left) were calculated by subtracting negative control values from mouse IgG spots (shown in the furthest panel to the right). *A*: monocyte-derived CD14^+^/CD81^+^ BAL EV were elevated in patients with sepsis-related ARDS compared with patients with sepsis without ARDS (medians 1.23 × 10^8^/mL vs. 6.26 × 10^7^/mL, *P* = 0.015). In esophagectomy patients, those who subsequently developed ARDS had lower CD14^+^/CD81^+^ BAL EV counts compared with patients who did not develop ARDS (medians 3.96 × 10^7^/mL vs. 9.94 × 10^7^/mL, *P* = 0.03). *B*: endothelial-derived CD31^+^/CD63^+^ and CD31^+^/CD81^+^ BAL EV were elevated in esophagectomy patients who subsequently developed ARDS compared with controls (*P* ≤ 0.02). *C*–*F*: there were no differences in the numbers of CD66b^+^, CD41^+^, CD206^+^, and EpCam^+^ EV between ARDS and control groups in both cohorts.

Characterization studies showed that a greater number of monocyte-derived CD14^+^/CD81^+^ EV were present in the BAL of patients with sepsis-related ARDS compared to patients with sepsis without ARDS (Fig. 2*A*, medians 1.23 × 10^8^/mL vs. 6.26 × 10^7^/mL, *P* = 0.015). However in esophagectomy patients, those who subsequently developed ARDS had lower CD14^+^/CD81^+^ BAL EV counts compared with patients who did not develop ARDS (Fig. 2*A*, medians 3.96 × 10^7^/mL versus 9.94 × 10^7^/mL, *P* = 0.03). Endothelial-derived CD31^+^/CD63^+^ and CD31^+^/CD81^+^ BAL EV were elevated in esophagectomy patients who subsequently developed ARDS compared with those who did not develop ARDS (Fig. 2*B*, *P* ≤ 0.02). There were no differences in the numbers of CD66b^+^, CD41^+^, CD206^+^, and EpCam^+^ BAL EV between ARDS and control groups in both cohorts (Fig. 2, *C*–*F*).

**Figure 3.**
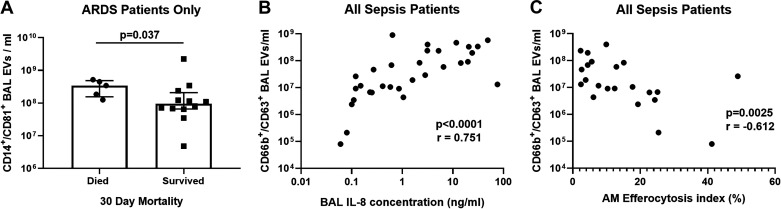
Correlations between bronchoalveolar lavage (BAL) extracellular vesicles (EV) and clinical, biochemical, and immune parameters. *A*: in patients with sepsis-related acute respiratory distress syndrome (ARDS), those who died within 30 days of intensive care unit (ICU) admission had a greater number of CD14^+^/CD81^+^ BAL EV than survivors (medians 3.43 × 10^8^/mL vs. 9.54 × 10^7^/mL, *P* = 0.027, *n* = 17). Statistical analysis by Mann–Whitney test, data shown as median and interquartile range. *B*: across all patients with sepsis, with and without ARDS, CD66b^+^/CD63^+^ BAL EV correlate directly with BAL interleukin (IL)-8 concentrations (*r* = 0.751, *P* < 0.0001, *n* = 31). Statistical analyses by Spearman’s correlation coefficient. *C*: across all patients with sepsis, with and without ARDS, CD66b^+^/CD63^+^ BAL EV correlate inversely with alveolar macrophage (AM) efferocytosis index (*r* = −0.612, *P* = 0.0025, *n* = 31). Statistical analyses by Spearman’s correlation coefficient.

In patients with sepsis-related ARDS, those who died within 30 days of intensive care unit (ICU) admission had a greater number of CD14^+^/CD81^+^ BAL EV than survivors (Fig. 3*A*, medians 3.43 × 10^8^/mL vs. 9.54 × 10^7^/mL, *P* = 0.027), however there is overlap between groups. Across all patients with sepsis, with and without ARDS, CD66b^+^/CD63^+^ BAL EV correlated directly with BAL interleukin (IL)-8 concentrations (Fig. 3*B*, *r* = 0.751, *P* < 0.0001). Across all patients with sepsis, with and without ARDS, CD66b^+^/CD63^+^ BAL EV correlated inversely with alveolar macrophage efferocytosis index (Fig. 3*C*, *r* = −0.612, *P* = 0.0025).

## DISCUSSION

In this study, patients with sepsis-related ARDS had significantly higher numbers of CD14^+^/CD81^+^ BAL EV than patients with sepsis without ARDS. CD14^+^/CD81^+^ BAL EV numbers were significantly higher in those patients with sepsis-related ARDS who died during the 30 days following ICU admission. CD206^+^ EV numbers were unchanged in patients with sepsis-related ARDS (CD206 being an alveolar macrophage marker), indicating that the increased CD14^+^ EV observed in patients with ARDS were derived from infiltrating monocytes as opposed to resident alveolar macrophages. CD14 has previously been used as a marker of monocyte-derived EV (18). The relationship between CD14^+^/CD81^+^ BAL EV and mortality in patients with sepsis-related ARDS is in keeping with previous studies showing that the degree of monocyte influx in ARDS can correlate with the severity of respiratory failure (19). Previous studies have shown that monocyte-derived plasma EV containing gasdermin D and activated caspase-1 are more abundant in patients with sepsis-related ARDS compared with healthy controls; uptake of these EV can induce human pulmonary vascular endothelial cell death (20). This data supports our hypothesis that CD14^+^ EV may contribute to ARDS pathogenesis, and might be utilized as a biomarker of disease severity. Within the sepsis-related ARDS cohort, there appear to be two distinct subpopulations of BAL CD14^+^ EV numbers: “high” and “low” (Fig. 2*A*). These may reflect different ARDS phenotypes (21), with the “high” CD14^+^ EV subpopulation indicating a hyper-inflammatory phenotype, and the “low” subpopulation indicating a hypoinflammatory phenotype; however further analysis of BAL fluid and physiological data would be required to confirm this. Conversely, CD14^+^/CD81^+^ BAL EV counts were decreased in esophagectomy patients who later developed ARDS compared with controls. BAL samples from esophagectomy patients were taken immediately postoperatively before the development of ARDS, compared with being taken at the time of ARDS diagnosis in patients with sepsis. This temporal difference in sampling and differences in the etiology of ARDS between the two cohorts may partially explain the divergent CD14^+^/CD81^+^ BAL EV counts. However, due to low *n* numbers in the postesophagectomy ARDS group, firm conclusions cannot be made and further appropriately powered studies are required to investigate the predictive role of CD14^+^/CD81^+^ BAL EV in esophagectomy patients.

We also observed the associations between CD66b^+^/CD63^+^ neutrophil-derived BAL EV in patients with sepsis and increased BAL IL-8 concentration and decreased alveolar macrophage efferocytosis. These data indicate that CD66b^+^/CD63^+^ BAL EV may be a marker of inflammation in sepsis-related ARDS. Neutrophil-derived EV have been shown to have an anti-inflammatory, tolerogenic effect following uptake by macrophages and T lymphocytes (22–25). Previous studies have also reported that neutrophil-derived EV can transfer microRNA-223 to alveolar epithelial cells, which reduces protein permeability and cytokine release via repression of poly[adenosine diphosphate-ribose] polymerase-1 (26). Therefore, the increased number of CD66b^+^ EV in patients with a more inflammatory alveolar microenvironment may be a compensatory protective response to suppress inflammation and reduce alveolar epithelial injury.

Our findings of increased CD31^+^/CD63^+^ and CD31^+^/CD81^+^ endothelial-derived EV in esophagectomy patients who subsequently developed ARDS may indicate an early signal of pulmonary endothelial injury resulting from one-lung ventilation detectable immediately postoperatively. This endothelial injury may initiate the development of ARDS in esophagectomy patients. Murine studies have shown that ventilator-induced lung injury can increase endothelial EV shedding (27); endothelial-derived EVs have been shown to induce acute lung injury by promoting neutrophil infiltration, endothelial dysfunction, and proinflammatory cytokine release (28, 29). Appropriately powered future studies are required to further investigate the role of CD31^+^ EV as a predictive biomarker in esophagectomy patients. No difference in CD31^+^ and CD41^+^ EVs were observed between patients with sepsis with and without ARDS. It is also important to note that the process of BAL dilutes the alveolar epithelial lining fluid by ∼100-fold, which may account for BAL EV numbers being lower than EV numbers detected in other biofluids.

EV transfer of microRNA cargo to murine macrophages can alter metabolic profile and function (30); EV transfer of long noncoding RNA can promote glycolysis (31). An association between impaired alveolar macrophage efferocytosis and dependence on glycolysis has been observed in patients with COPD (32). Further studies are required to determine if similar pathological processes occur in ARDS, specifically whether CD14^+^ EV transfer of microRNA or protein cargo induces alveolar macrophage dysfunction (12), thereby promoting persistent inflammation. Understanding the role of EV in ARDS pathogenesis will allow identification of potential EV/microRNA biomarkers and novel therapeutic targets for ARDS.

A limitation of our study is that while we identified EV from the major cell types present in the alveolar space, not all cellular origins were included in our analysis (e.g., dendritic cells and lymphocytes). Although tetraspanin capture of EVs provides greater specificity compared with other isolation methods, it also limits phenotyping, and therefore only provides an indication of the relevance of CD14^+^ EV. Another limitation is that we did not capture EV based on CD14^+^, CD31^+^, and CD66b^+^ expression, which would allow probing of external markers for extensive phenotyping. Future studies are required to undertake this and probe for internal markers (e.g., syntenin) to determine EV biogenesis pathways. The finding that CD14 most commonly colocalizes with tetraspanin CD81, and CD66b with tetraspanin CD63, may indicate different EV biogenesis pathways in monocytes and neutrophils (33). A further limitation of our study is that the observed differences in CD14^+^ BAL EVs were modest, and it is unclear whether these differences are biologically relevant. Similarly, correlations observed with CD14^+^, CD31^+^, and CD66b^+^ BAL EVs cannot be used to draw conclusions on their functional roles. Further work is required to determine the biological function of CD14^+^ and CD31^+^ BAL EV subpopulations in patients with ARDS. Another limitation of our study is that the number of VINDALOO trial patients who received placebo and developed ARDS following esophagectomy was low (*n* = 4), thus preventing us from drawing firm conclusions regarding the role of EVs as biomarkers in this population.

In conclusion, we report that CD14^+^/CD81^+^ BAL EV are enriched in patients with sepsis-related ARDS compared with controls, and that an elevated CD14^+^/CD81^+^ BAL EV count is associated with increased mortality in patients with ARDS, although the sample size is modest. Thus, CD14^+^ BAL EV are a potential biomarker for disease severity and mortality in sepsis-related ARDS. Our findings provide the impetus to further elucidate the role these EV play in ARDS pathogenesis.

## ETHICAL APPROVAL

Ethical approval was obtained to recruit ventilated sepsis patients with and without ARDS (REC 16/WA/0169). Ethical approval was also obtained to recruit patients undergoing transthoracic esophagectomy for esophageal carcinoma (REC 12/WM/0092) as previously described in the VINDALOO trial. For patients who lacked capacity, permission to enroll was sought from a personal legal representative in accordance with the UK Mental Capacity Act (2005). For patients with capacity, written informed consent was obtained from the patient.

## GRANTS

This work was funded by Medical Research Council Grants MR/N021185/1 (to R. Y. Mahida) and MR/L002736/1 (to D. R. Thickett and A. Scott), and by National Heart, Lung, and Blood Institute Grants HL134828 (to M. A. Matthay) and HL140026 (to M. A. Matthay). The ExoView platform was funded by an EPSRC Capital award awarded to Dr. Sophie Cox, University of Birmingham.

## DISCLOSURES

No conflicts of interest, financial or otherwise, are declared by the authors.

## AUTHOR CONTRIBUTIONS

R.Y.M., D.P., A.S., P.H., M.A.M., and D.R.T. conceived and designed research; R.Y.M., J.P., S.T.L., and H.L. performed experiments; R.Y.M. and J.P. analyzed data; R.Y.M., J.P., P.H., and M.A.M. interpreted results of experiments; R.Y.M. prepared figures; R.Y.M. and J.P. drafted manuscript; R.Y.M., J.P., S.T.L., H.L., D.P., A.S., P.H., M.A.M., and D.R.T. edited and revised manuscript; R.Y.M., J.P., S.T.L., H.L., D.P., A.S., P.H., M.A.M., and D.R.T. approved final version of manuscript.

## References

[1] Matthay MA, Zemans RL, Zimmerman GA, Arabi YM, Beitler JR, Mercat A, Herridge M, Randolph AG, Calfee CS. Acute respiratory distress syndrome. Nat Rev Dis Primers 5: 18, 2019. doi:10.1038/s41572-019-0069-0. 30872586 PMC6709677

[2] Bellani G, Laffey JG, Pham T, Fan E, Brochard L, Esteban A, Gattinoni L, van Haren F, Larsson A, McAuley DF, Ranieri M, Rubenfeld G, Thompson BT, Wrigge H, Slutsky AS, Pesenti A; ESICM Trials Group. Epidemiology, patterns of care, and mortality for patients with acute respiratory distress syndrome in intensive care units in 50 countries. JAMA 315: 788–800, 2016. doi:10.1001/jama.2016.0291. 26903337

[3] Yang X, Yu Y, Xu J, Shu H, Xia J, Liu H, Wu Y, Zhang L, Yu Z, Fang M, Yu T, Wang Y, Pan S, Zou X, Yuan S, Shang Y. Clinical course and outcomes of critically ill patients with SARS-CoV-2 pneumonia in Wuhan, China: a single-centered, retrospective, observational study. Lancet Respir Med 8: 475–481, 2020 [Erratum in *Lancet Respir Med* 8: e26, 2020]. doi:10.1016/S2213-2600(20)30079-5. 32105632 PMC7102538

[4] Mahida RY, Matsumoto S, Matthay MA. Extracellular vesicles: a new frontier for research in acute respiratory distress syndrome. Am J Respir Cell Mol Biol 63: 15–24, 2020. doi:10.1165/rcmb.2019-0447TR. 32109144 PMC7328246

[5] Shah R, Patel T, Freedman JE. Circulating extracellular vesicles in human disease. N Engl J Med 379: 958–966, 2018. doi:10.1056/NEJMra1704286. 30184457

[6] Gupta R, Radicioni G, Abdelwahab S, Dang H, Carpenter J, Chua M, Mieczkowski PA, Sheridan JT, Randell SH, Kesimer M. Intercellular communication between airway epithelial cells is mediated by exosome-like vesicles. Am J Respir Cell Mol Biol 60: 209–220, 2019. doi:10.1165/rcmb.2018-0156OC. 30230353 PMC6376407

[7] Khan NZ, Cao T, He J, Ritzel RM, Li Y, Henry RJ, Colson C, Stoica BA, Faden AI, Wu J. Spinal cord injury alters microRNA and CD81^+^ exosome levels in plasma extracellular nanoparticles with neuroinflammatory potential. Brain Behav Immun 92: 165–183, 2021. doi:10.1016/j.bbi.2020.12.007. 33307173 PMC7897251

[8] Kugeratski FG, Hodge K, Lilla S, McAndrews KM, Zhou X, Hwang RF, Zanivan S, Kalluri R. Quantitative proteomics identifies the core proteome of exosomes with syntenin-1 as the highest abundant protein and a putative universal biomarker. Nat Cell Biol 23: 631–641, 2021. doi:10.1038/s41556-021-00693-y. 34108659 PMC9290189

[9] Liu A, Park JH, Zhang X, Sugita S, Naito Y, Lee JH, Kato H, Hao Q, Matthay MA, Lee JW. Therapeutic effects of hyaluronic acid in bacterial pneumonia in the ex vivo perfused human lungs. Am J Respir Crit Care Med 200: 1234–1245, 2019. doi:10.1164/rccm.201812-2296OC. 31390880 PMC6857490

[10] Shikano S, Gon Y, Maruoka S, Shimizu T, Kozu Y, Iida Y, Hikichi M, Takahashi M, Okamoto S, Tsuya K, Fukuda A, Mizumura K, Hashimoto S. Increased extracellular vesicle miRNA-466 family in the bronchoalveolar lavage fluid as a precipitating factor of ARDS. BMC Pulm Med 19: 110, 2019. doi:10.1186/s12890-019-0876-9. 31221118 PMC6584994

[11] Bazzan E, Radu CM, Tinè M, Neri T, Biondini D, Semenzato U, Casara A, Balestro E, Simioni P, Celi A, Cosio MG, Saetta M. Microvesicles in bronchoalveolar lavage as a potential biomarker of COPD. Am J Physiol Lung Cell Mol Physiol 320: L241–L245, 2021. doi:10.1152/ajplung.00362.2020. 33146565

[12] Mahida RY, Scott A, Parekh D, Lugg ST, Hardy RS, Lavery GG, Matthay MA, Naidu B, Perkins GD, Thickett DR. Acute respiratory distress syndrome is associated with impaired alveolar macrophage efferocytosis. Eur Respir J 58: 2100829, 2021. doi:10.1183/13993003.00829-2021. 34112730 PMC8754102

[13] Mahida RY, Scott A, Parekh D, Lugg ST, Belchamber KB, Hardy RS, Matthay MA, Naidu B, Thickett DR. Assessment of alveolar macrophage dysfunction using an in vitro model of acute respiratory distress syndrome. Front Med (Lausanne) 8: 737859, 2021. doi:10.3389/fmed.2021.737859. 34660643 PMC8511446

[14] Parekh D, Dancer RC, Lax S, Cooper MS, Martineau AR, Fraser WD, Tucker O, Alderson D, Perkins GD, Gao-Smith F, Thickett DR. Vitamin D to prevent acute lung injury following oesophagectomy (VINDALOO): study protocol for a randomised placebo controlled trial. Trials 14: 100, 2013. doi:10.1186/1745-6215-14-100. 23782429 PMC3680967

[15] Parekh D, Dancer RCA, Scott A, D'Souza VK, Howells PA, Mahida RY, Tang JCY, Cooper MS, Fraser WD, Tan L, Gao F, Martineau AR, Tucker O, Perkins GD, Thickett DR. Vitamin D to prevent lung injury following esophagectomy—a randomized, placebo-controlled trial. Crit Care Med 46: e1128–e1135, 2018. doi:10.1097/CCM.0000000000003405. 30222631 PMC6250246

[16] Haslam PL, Baughman RP. Report of ERS task force: guidelines for measurement of acellular components and standardization of BAL. Eur Respir J 14: 245–248, 1999. doi:10.1034/j.1399-3003.1999.14b01.x. 10515395

[17] Price J, Gardiner C, Harrison P. Platelet-enhanced plasma: characterization of a novel candidate resuscitation fluid’s extracellular vesicle content, clotting parameters, and thrombin generation capacity. Transfusion 61: 2179–2194, 2021. doi:10.1111/trf.16423. 33948950

[18] Wang GH, Lu J, Ma KL, Zhang Y, Hu ZB, Chen PP, Lu CC, Zhang XL, Liu BC. The release of monocyte-derived tissue factor-positive microparticles contributes to a hypercoagulable state in idiopathic membranous nephropathy. J Atheroscler Thromb 26: 538–546, 2019. doi:10.5551/jat.46284. 30429407 PMC6545459

[19] Rosseau S, Hammerl P, Maus U, Walmrath HD, Schütte H, Grimminger F, Seeger W, Lohmeyer J. Phenotypic characterization of alveolar monocyte recruitment in acute respiratory distress syndrome. Am J Physiol Lung Cell Mol Physiol 279: L25–L35, 2000. doi:10.1152/ajplung.2000.279.1.L25. 10893199

[20] Mitra S, Exline M, Habyarimana F, Gavrilin MA, Baker PJ, Masters SL, Wewers MD, Sarkar A. Microparticulate caspase 1 regulates gasdermin D and pulmonary vascular endothelial cell injury. Am J Respir Cell Mol Biol 59: 56–64, 2018. doi:10.1165/rcmb.2017-0393OC. 29365280 PMC6039876

[21] Calfee CS, Delucchi K, Parsons PE, Thompson BT, Ware LB, Matthay MA. Subphenotypes in acute respiratory distress syndrome: latent class analysis of data from two randomised controlled trials. Lancet Respir Med 2: 611–620, 2014. doi:10.1016/S2213-2600(14)70097-9. 24853585 PMC4154544

[22] Shen G, Krienke S, Schiller P, Nießen A, Neu S, Eckstein V, Schiller M, Lorenz HM, Tykocinski LO. Microvesicles released by apoptotic human neutrophils suppress proliferation and IL-2/IL-2 receptor expression of resting T helper cells. Eur J Immunol 47: 900–910, 2017. doi:10.1002/eji.201546203. 28295230

[23] Eken C, Martin PJ, Sadallah S, Treves S, Schaller M, Schifferli JA. Ectosomes released by polymorphonuclear neutrophils induce a MerTK-dependent anti-inflammatory pathway in macrophages. J Biol Chem 285: 39914–39921, 2010. doi:10.1074/jbc.M110.126748. 20959443 PMC3000973

[24] Gasser O, Schifferli JA. Activated polymorphonuclear neutrophils disseminate anti-inflammatory microparticles by ectocytosis. Blood 104: 2543–2548, 2004. doi:10.1182/blood-2004-01-0361. 15213101

[25] Dengler V, Downey GP, Tuder RM, Eltzschig HK, Schmidt EP. Neutrophil intercellular communication in acute lung injury. Emerging roles of microparticles and gap junctions. Am J Respir Cell Mol Biol 49: 1–5, 2013. doi:10.1165/rcmb.2012-0472TR. 23815257 PMC3727882

[26] Neudecker V, Brodsky KS, Clambey ET, Schmidt EP, Packard TA, Davenport B, Standiford TJ, Weng T, Fletcher AA, Barthel L, Masterson JC, Furuta GT, Cai C, Blackburn MR, Ginde AA, Graner MW, Janssen WJ, Zemans RL, Evans CM, Burnham EL, Homann D, Moss M, Kreth S, Zacharowski K, Henson PM, Eltzschig HK. Neutrophil transfer of miR-223 to lung epithelial cells dampens acute lung injury in mice. Sci Transl Med 9: eaah5360, 2017. doi:10.1126/scitranslmed.aah5360. 28931657 PMC5842431

[27] Letsiou E, Sammani S, Zhang W, Zhou T, Quijada H, Moreno-Vinasco L, Dudek SM, Garcia JG. Pathologic mechanical stress and endotoxin exposure increases lung endothelial microparticle shedding. Am J Respir Cell Mol Biol 52: 193–204, 2015. doi:10.1165/rcmb.2013-0347OC. 25029266 PMC4370243

[28] Buesing KL, Densmore JC, Kaul S, Pritchard KA Jr, Jarzembowski JA, Gourlay DM, Oldham KT. Endothelial microparticles induce inflammation in acute lung injury. J Surg Res 166: 32–39, 2011. doi:10.1016/j.jss.2010.05.036. 20828748 PMC4731030

[29] Densmore JC, Signorino PR, Ou J, Hatoum OA, Rowe JJ, Shi Y, Kaul S, Jones DW, Sabina RE, Pritchard KA Jr, Guice KS, Oldham KT. Endothelium-derived microparticles induce endothelial dysfunction and acute lung injury. Shock 26: 464–471, 2006. doi:10.1097/01.shk.0000228791.10550.36. 17047516

[30] Park JE, Dutta B, Tse SW, Gupta N, Tan CF, Low JK, Yeoh KW, Kon OL, Tam JP, Sze SK. Hypoxia-induced tumor exosomes promote M2-like macrophage polarization of infiltrating myeloid cells and microRNA-mediated metabolic shift. Oncogene 38: 5158–5173, 2019. doi:10.1038/s41388-019-0782-x. 30872795

[31] Chen F, Chen J, Yang L, Liu J, Zhang X, Zhang Y, Tu Q, Yin D, Lin D, Wong PP, Huang D, Xing Y, Zhao J, Li M, Liu Q, Su F, Su S, Song E. Extracellular vesicle-packaged HIF-1α-stabilizing lncRNA from tumour-associated macrophages regulates aerobic glycolysis of breast cancer cells. Nat Cell Biol 21: 498–510, 2019. doi:10.1038/s41556-019-0299-0. 30936474

[32] Ryan E, Coelho P, Cole J, Bewley M, Budd R, Callahan J, McCafferty J, Singh D, Dockrell D, Walmsley S, Whyte M. T1 defective metabolism drives macrophage dysfunction in COPD (Abstract). Thorax 76: A1, 2021. doi:10.1136/thorax-2020-BTSabstracts.1.

[33] Mathieu M, Névo N, Jouve M, Valenzuela JI, Maurin M, Verweij FJ, Palmulli R, Lankar D, Dingli F, Loew D, Rubinstein E, Boncompain G, Perez F, Théry C. Specificities of exosome versus small ectosome secretion revealed by live intracellular tracking of CD63 and CD9. Nat Commun 12: 4389, 2021. doi:10.1038/s41467-021-24384-2. 34282141 PMC8289845

